# Absence of TAAR1 function increases methamphetamine-induced excitability of dorsal raphe serotonin neurons and drives binge-level methamphetamine intake

**DOI:** 10.1038/s41386-025-02063-w

**Published:** 2025-02-11

**Authors:** Samantha M. Rios, John R. K. Mootz, Tamara J. Phillips, Susan L. Ingram

**Affiliations:** 1 Department of Behavioral Neuroscience, Oregon Health & Science University, Portland, OR USA; 2 Veterans Affairs Portland Health Care System, Portland, OR USA; 3 Department of Anesthesiology, University of Colorado Anschutz Medical Campus, Aurora, CO USA

**Keywords:** Action potential generation, Behavioural genetics

## Abstract

Methamphetamine (MA) is a potent psychostimulant capable of exerting both rewarding and aversive effects, the balance of which likely drives variation in voluntary MA intake. Understanding the genetic factors underlying sensitivity to these effects of MA is critical for developing effective treatments. The activity of dorsal raphe serotonin neurons is linked to reward processing. Here, we performed whole-cell patch-clamp electrophysiology in dorsal raphe serotonin neurons from mice with high or low MA intake corresponding with high or low MA reward sensitivity. The MA drinking (MADR) mice consist of the MA reward sensitive MA high drinking (MAHDR) and the MA reward insensitive MA low drinking (MALDR) lines. MA is a trace amine-associated receptor 1 (TAAR1) agonist, and MAHDR mice are homozygous for a mutation in the *Taar1* gene*, Taar1*^*m1J*^, that encodes non-functional TAAR1, whereas MALDR mice possess at least one copy of the reference *Taar1*^*+*^ allele that encodes functional TAAR1. Our previous research using CRISPR-*Cas9*-generated MAHDR-*Taar1*^*+/+*^ knock-in mice in which *Taar1*^*m1J*^ was replaced with *Taar1*^*+*^, and non-edited MAHDR-*Taar1*^*m1J/m1J*^ controls demonstrated that lack of TAAR1 function is critical for heightened MA consumption and MA reward sensitivity. Here, electrophysiological recordings in the MADR lines demonstrate a MA-induced decrease in dorsal raphe serotonin neuron activity from MALDR, but not MAHDR mice. However, in the presence of serotonin autoreceptor antagonists, MA potentiates dorsal raphe serotonin neuron activity of MAHDR, but not MALDR mice. Importantly, potentiation in the presence of the antagonists is abolished in knock-in mice expressing functional TAAR1. The knock-in mice did not display binge-level MA intake, consistent with the loss of MA-reward sensitivity previously reported in mice with functional TAAR1. Finally, because MA is a substrate of the serotonin transporter, we evaluated whether the serotonin transporter is necessary for MA-induced potentiation of dorsal raphe serotonin neuron activity in mice with non-functional TAAR1. The serotonin transporter antagonist fluoxetine blocks MA-induced potentiation for both MAHDR and MAHDR-*Taar1*^*m1J/m1J*^ mice. Thus, TAAR1 function directly impacts MA reward sensitivity and MA intake and serves as a critical regulator of MA-induced activity of dorsal raphe serotonin neurons through its interaction with the serotonin transporter.

## Introduction

The incidence of methamphetamine (MA) use disorders has dramatically risen during the past decade [1]. No effective treatments for MA use disorder exist. Further investigation into mechanisms by which MA influences neurotransmission in brain regions associated with reward and aversion processing is necessary for understanding cellular mechanisms underlying addiction and for the development of MA use disorder therapies. A vigorous genetic tool for examining the impact of initial sensitivity to rewarding and aversive effects of MA on subsequent MA use is mice selectively bred for differential voluntary MA consumption. Our lab employed a two-bottle choice voluntary MA consumption procedure to generate the MA drinking (MADR) selected lines, consisting of mice bred for high and low MA intake; the MA high drinking (MAHDR) and MA low drinking (MALDR) lines, respectively [2]. Behaviorally, MAHDR mice exhibit high sensitivity to the rewarding effects of MA, while MALDR mice exhibit insensitivity. High reward sensitivity in MAHDR mice is associated with diminished sensitivity to the aversive effects of MA, compared to MALDR mice, which exhibit high aversion sensitivity. Selection response for high vs. low MA consumption and differential sensitivity to rewarding and aversive effects of MA have been confirmed across five replicate sets of MADR lines [2–5].

Whole genome mapping identified a location on mouse chromosome 10 accounting for 60% of the genetic variance in MA intake between the MADR lines [6], which was traced to a single nucleotide polymorphism within the coding sequence of the trace amine-associated receptor 1 (*Taar1)* gene [7, 8]. MA is a full agonist at the intracellular G protein-coupled receptor encoded by the *Taar1* gene, TAAR1 [9]. Within 1-2 generations of selective breeding, all MAHDR mice are homozygous for the spontaneously mutated *Taar1* allele, denoted as *Taar1*^*m1J*^, that encodes non-functional TAAR1 [10]. Conversely, MALDR mice possess at least one copy of the reference *Taar1*^*+*^ allele that encodes functional TAAR1 [10]. Using a CRISPR-*Cas9*-generated MAHDR*-Taar1*^+/+^ knock-in (KI) line, compared to a MAHDR-*Taar1*^*m1J/m1J*^ line that served as a control for the KI, TAAR1 functionality was determined to be critical for differential MA intake, and sensitivity to rewarding, aversive, and physiological effects of MA [8, 11]. Herein, we used the MAHDR*-Taar1*^+/+^ KI and MAHDR-*Taar1*^*m1J/m1J*^ mice to examine whether replacement with functional TAAR1 would attenuate binge-level MA intake, as it does for MA intake at low concentrations [8]. Mice were tested using a two-bottle choice procedure with increasing MA concentrations previously used to demonstrate binge-level MA intake in MAHDR mice [12].

Overall, the pronounced imbalance between MA-induced reward and aversion sensitivity in the MADR mice, linked to TAAR1 functionality, makes them ideal for determining whether the effects of MA on neural activity (1) correspond with the perception of MA reward or aversion and (2) are dependent on TAAR1 functionality. Our studies focus on the dorsal raphe (DR) which contains serotonin (5-HT) neurons. DR 5-HT neurons are involved in the perception of rewarding stimuli, including sucrose, food, and social interaction [13–17]. DR 5-HT neuron activity increases in response to rewarding stimuli [13–15] and optogenetic stimulation of DR 5-HT neurons induces reward-motivated behaviors [13, 16, 17]. In contrast, optogenetic inhibition of DR 5-HT neurons reduces reward-motivated behavior [17]. Amphetamines increase 5-HT release in the DR [18, 19] and TAAR1 is widely expressed within this region [20–22]. Activation of Gα_13_-coupled TAAR1 by the psychostimulant MDMA stimulates RhoA, leading to the internalization of the serotonin transporter (SERT) and a decrease in 5-HT uptake in a TAAR1 function-dependent manner [23]. In response to the TAAR1 agonist, RO5166017, DR 5-HT neurons exhibit a reduction in firing, an effect that is absent in DR 5-HT TAAR1 KO cells [24]. These studies demonstrate that TAAR1 modulates 5-HT concentrations and DR 5-HT neuron activity.

To investigate the relationship between TAAR1 function and DR 5-HT neuron activity in mice with differential MA intake and sensitivity to MA-induced reward, we performed whole-cell patch clamp electrophysiology experiments on DR 5-HT neurons from MADR and CRISPR-*Cas9* generated mice. By examining the electrophysiological properties of these neurons, we determined the influence of TAAR1 functionality and underlying mechanisms of MA’s effects on intrinsic neuronal activity of DR 5-HT neurons.

## Materials and Methods

### Animal maintenance and housing

All mice were born within the VA Portland Health Care System (VAPORHCS) veterinary medical unit. After weaning, mice were maintained in standard acrylic plastic shoebox cages on corncob bedding with wire lids and filter tops. Mice were maintained in climate-controlled rooms under a standard 12:12 h light: dark cycle with lights on at 0600 h and *ad libitum* access to water and rodent block food (5LOD PicoLab Rodent Diet; Animal Specialties, Woodburn, Oregon). All animal care and testing procedures were approved by the VAPORHCS Animal Care and Use Committee and were conducted in compliance with the National Institutes of Health Guidelines for Care and Use of Laboratory Animals.

### Methamphetamine drinking selected mouse lines

MA-naïve male and female MAHDR and MALDR mice 28-45 days of age were used for electrophysiological studies. MADR mice were selectively bred from a reciprocal F2 cross of C57BL/J6 and DBA/2J inbred strains, based on voluntary MA intake during a two-bottle choice procedure. Details of the selective breeding procedures and responses to the selection of multiple replicate sets of MADR lines have been fully described in previous publications [2, 3, 5]. Briefly, mice were provided a water bottle versus 20 mg/L MA in water for 18 h/day for 4 days and then 40 mg/L MA in water for an additional 4 days. Mice used for selective breeding were chosen based on average MA consumed in mg/kg, during access to the 40 mg/L MA solution.

### CRISPR-*Cas9* knock-in of *Taar1*^*+*^

Male and female MAHDR-*Taar1*^*+/+*^ KI and MAHDR-*Taar1*^*m1J/m1J*^ mice 82-85 days of age were tested in an escalating MA concentration two-bottle choice procedure and mice 29-43 days of age were used for electrophysiological study. The MAHDR*-Taar1*^*+/+*^ KI mice were created at Oregon Health & Science University’s Transgenic Mouse Models Shared Resource Core, utilizing CRISPR-*Cas9* technology to exchange the *Taar1*^*m1J*^ allele with the *Taar1*^*+*^ reference allele. The MAHDR-*Taar1*^*m1J/m1J*^ line that served as a control for the KI was derived from mice in which the *Taar1*^*m1J*^ allele was not successfully excised and exchanged, thus retaining the *Taar1*^*m1J/m1J*^ genotype. Further details can be found in Stafford et al. [8].

### Drugs

(+) MA hydrochloride was purchased from Sigma-Aldrich (St. Louis, MO, USA). D-APV, bicuculline, and fluoxetine were purchased from HelloBio (Princeton, NJ, USA). SB 216641 and WAY 100635 were purchased from Cayman Chemical (Ann Arbor, MI, USA). Serotonin hydrochloride was purchased from Sigma Aldrich (Burlington, MA, USA). All drugs were dissolved in double distilled water, except when MA was used for drinking when it was dissolved in tap water.

### Two-bottle choice drinking of escalating MA concentrations

Methods were consistent with our previous study [12]. Voluntary MA consumption was measured from 20 to 140 mg/L MA concentrations, with concentration increasing in 20 mg/L increments every 4 days. Forty mice (10 per MAHDR-*Taar1*^*+/+*^ KI and MAHDR-*Taar1*^*m1J/m1J*^ line per sex) were weighed and individually housed in plastic shoe box cages with stainless steel wire tops. For the first 48 hours, mice acclimated to consuming fluid from the novel drinking bottles, 25-ml graduated cylinders fitted with stoppers and stainless-steel sippers placed between bars of the cage tops. Food and one water bottle were provided *ad libitum* during this period. On day 3, mice were weighed, and MA-containing bottles were added onto the cage tops for an 18-h period 3-h before the dark cycle started and removed 3-h into the light phase. Fluid consumption was determined for both the 18-h (water vs. MA) and 6-h (water only) periods. To account for position bias, the position of water and MA bottles was alternated every 2 days. Body weight data were collected every 2 days. Fluid consumption and body weight data were used to determine mg/kg of MA consumed daily. Consistent with selection and previous studies, mg/kg consumed during days 2 and 4 (the second day after a water vs. MA bottle position switch) of each MA concentration were averaged to represent drinking for each concentration.

### Brain slice preparation and electrophysiological recordings

Mice were deeply anesthetized with isoflurane for brain removal. Brains were immersed in ice-cold sucrose aCSF containing the following (in mM): 80 NaCl, 2.7 KCl, 0.1 CaCl_2_, 6.5 MgSO_4_, 1.3 NaH_2_PO_4_, 24 NaHCO_3_, 2.8 dextrose, and 82 sucrose with 87.5 µM D-APV, equilibrated with 95.0% O_2_/5% CO_2_. Coronal slices containing the DR were cut 230–250 µm thick with a vibratome (Leica Microsystems) and placed in oxygenated aCSF containing the following (in mM): 123.5 NaCl, 21 NaHCO_3_, 19 dextrose, 2.45 KCl, 2.55 CaCl_2_, 1.2 MgSO_4_, and 1.2 NaH_2_PO_4_, and equilibrated with 95% O_2_/5% CO_2_ at 34 °C until the start of recording. Brain slices were placed onto the recording chamber on an upright Olympus BX51WI microscope and superfused with 31–33 °C aCSF. Electrophysiological recordings were made using the Sutter Instruments Integrated Patch Clamp Amplifier and data acquisition system (Sutter Instruments, Novato, CA, USA). Data were acquired at 5 kHz and low pass filtered at 2 kHz.

Whole-cell recordings in current clamp mode were conducted with glass electrodes with resistances of 3 - 6 MΩ and filled with potassium gluconate internal solution containing the following (in mM): 127 D-gluconic acid potassium salt, 10 HEPES, 1 EGTA, 10 KCl, 1 MgCl_2_, 0.3 CaCl_2_, 2 MgATP, and 0.5 NaGTP, pH 7.3–7.4, and 285–295 mOsm. A junction potential of 15 mV was corrected at the start of experiments and for all reported resting membrane potentials (RMPs). During whole-cell current clamp experiments, no holding current was applied. Only neurons with stable RMPs that exhibited action potentials crossing 0 mV when depolarized by current step protocols were used for analysis. In the current clamp mode, 2 s long depolarizing steps (−40 pA to +60 pA in 20 pA increments, every 10 s) were used to evaluate the firing patterns of DR 5HT neurons. RMP was measured during the 100 ms before the current injection.

Putative serotonergic DR neurons were selected initially by their reversible inhibitory response to bath application of serotonin hydrochloride (10 µM). Neurons with a capacitance exceeding 50 pF were confirmed as serotonergic and subsequently used for electrophysiological studies.

### Experimental design and statistical analysis

All firing frequency data are expressed as mean ± SEM. Data were analyzed using Statistica 13.3 software (TIBCO Software, Inc, Palo Alto, CA, USA). Each cell is considered an independent observation; numbers of cells and mice are given in the figure legends. Differences in firing frequency were assessed using repeated measures ANOVA, followed by Tukey HSD when appropriate. Differences in RMPs between the same cells were assessed using paired t-tests, while comparisons between different cells were analyzed using unpaired t-tests. Differences in MA and total consumption were assessed using repeated measures ANOVA, followed by within-subjects or between mouse lines contrasts of means when appropriate. The level of significance for all statistical tests was set at ≤0.05.

## Results

### MA hyperpolarizes and inhibits the firing of MALDR DR 5-HT neurons

**Fig. 1:**
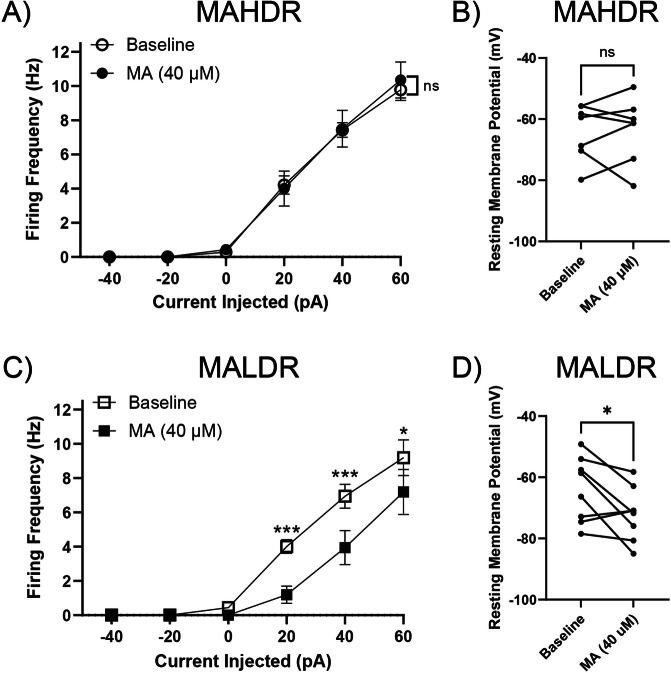
MA hyperpolarizes and inhibits firing of DR 5-HT neurons from MALDR mice. **A** Mean firing frequency of MAHDR DR 5-HT neurons pre- and post-MA application [7 recordings (7 mice: Male=3, Female=4). Means ± SEM are presented collapsed on sex.]. **B** RMPs of MAHDR DR 5-HT neurons were not significantly changed by MA. Each set of symbols represents a recording in the absence and presence of MA. **C** Mean firing frequency of MALDR DR 5-HT neurons pre- and post-MA application [8 recordings (8 mice: Male=4, Female=4); Tukey’s HSD post hoc test, *p < 0.05, ***p < 0.001 for baseline compared to MA at a given current. Means ± SEM are presented collapsed on sex.] **D** RMPs of MALDR DR 5-HT neurons were significantly hyperpolarized by MA, *p < 0.05). Each set of symbols represents a recording in the absence and presence of MA.

TAAR1 agonists inhibit monoamine neurons, including DR 5-HT neurons from C57BL/6J mice [24, 25], a MADR progenitor strain with the *Taar1* gene variant that encodes functional TAAR1 [7]. In contrast, TAAR1 agonists have no effect on DR 5-HT neuron activity in TAAR1 KO mice [24]. To determine the effects of MA on DR 5-HT neuron activity in brain slices from the MADR mouse lines, we measured spontaneous firing frequency and firing frequency across a series of current injections pre- and post-MA application (Fig. 1). MA superfusion alone did not affect the mean firing frequency of DR 5-HT neurons of MAHDR mice, which possess non-functional TAAR1 (Fig. 1A; treatment: *F*_(1,6)_ = 0.05, *p* = 0.83). MA had no effect on the RMPs of MAHDR DR 5-HT neurons (Fig.1B; *t*_(6)_ = 0.22, *p* = 0.84). However, MA superfusion significantly decreased the mean firing frequency of DR 5-HT neurons of MALDR mice, which possess functional TAAR1 (Fig. 1C; treatment: *F*_(1,7)_ = 12.46, *p* = 0.0096; treatment x current injected: *F*_(5,35)_ = 8.01, *p* = <0.001). Furthermore, MA significantly hyperpolarized MALDR DR 5-HT neurons (Fig. 1D; *t*_(7)_ = 2.57, *p* = 0.037).

### MA potentiates DR 5-HT neuron activity of MAHDR but not MALDR mice in the presence of 5HT autoreceptor antagonists

**Fig. 2:**
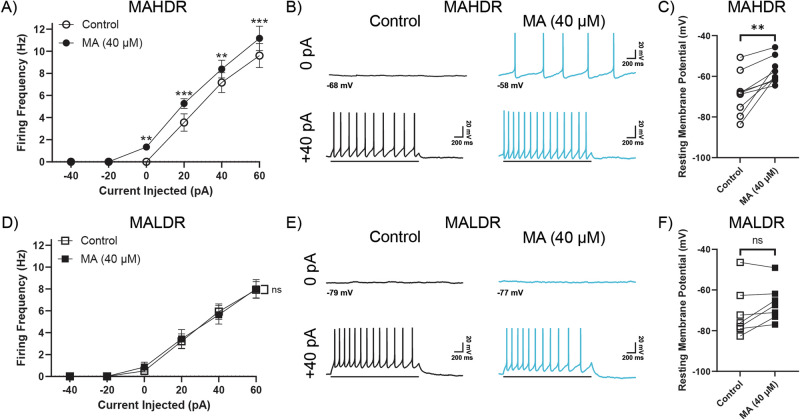
MA potentiates DR 5-HT neuron activity of MAHDR but not MALDR mice in the presence of 5HT_1A_ and 5HT_1B_ autoreceptor antagonists. Control refers to the firing frequency, activity, and RMP in the presence of antagonists for 5HT_1A_ (WAY 100635, 100 nM) and 5HT_1B_ (SB 216641, 200 nM) autoreceptors. **A** Mean firing frequency of MAHDR DR 5-HT neurons pre- and post-MA application [9 recordings (8 mice: Male = 5, Female = 4); Tukey’s HSD post hoc test, **p < 0.01, ***p < 0.001 for control compared to MA at a given current. Means ± SEM are presented collapsed on sex.] **B** Representative traces of MAHDR DR 5-HT recordings at 0 pA and +40 pA current injection in the absence (black) and presence of MA (teal). **C** RMPs of MAHDR DR 5-HT neurons were significantly depolarized by MA, **p < 0.01). Each set of symbols represents a recording in the absence and presence of MA. **D** Mean firing frequency of MALDR DR 5-HT neurons pre- and post-MA application [7 recordings (7 mice: Male = 3, Female = 4). Means ± SEM are presented collapsed on sex.] **E** Representative traces of MALDR DR 5-HT recordings at 0 pA and +40 pA current injection in the absence (black) and presence of MA (teal). **F** RMPs of MALDR DR 5-HT neurons were not significantly changed by MA. Each set of symbols represents a recording in the absence and presence of MA.

MA is a substrate of SERT [26] and increases extracellular 5-HT concentrations by competing for intracellular transport. Increased extracellular 5-HT can induce feedback inhibition of DR 5-HT neurons through activation of 5-HT_1A_ and 5-HT_1B_ autoreceptors [27, 28]. To determine whether MA alters intrinsic activity of DR 5-HT neurons, spontaneous firing, and firing frequency across a series of current injections were recorded pre- and post-MA application in the presence of 5HT_1A_ and 5HT_1B_ autoreceptor antagonists. MA significantly increased the overall firing frequency of MAHDR DR 5-HT neurons (Fig. 2A; treatment: *F*_(1,8)_ = 29.70, *p* = <0.001; treatment x current injected: *F*_(5,40)_ = 7.03, *p* = <0.001). Representative traces demonstrate that MA potentiated MAHDR DR 5-HT neuron activity when recording spontaneous activity and when injecting +40 pA of current (Fig. 2B). MA depolarized the RMPs of MAHDR DR 5-HT neurons (Fig. 2C; *t*_(8)_ = 4.36, *p* = 0.0024). We observed a different profile in MALDR mice, where MA had no effect in the presence of 5-HT autoreceptor inhibitors (Fig. 2D; treatment: *F*_(1,6)_ = 0.26, *p* = 0.63). Representative traces demonstrate that MA had no effect on spontaneous firing or firing during the +40 pA current injection (Fig. 2E) and no effect on RMPs (Fig. 2F; *t*_(6)_ = 2.21, *p* = 0.07) of MALDR DR 5-HT neurons. These data indicate that the MA-induced reduction in firing and hyperpolarization observed in MALDR mice (Fig. 1C, D) is due to the activation of 5-HT autoreceptors.

### Binge-level MA consumption is dependent on *Taar1*^*m1J*^ encoding non-functional TAAR1

Our previous work first linked [7, 10], then determined a causal role [8, 11], for TAAR1 functionality in determining differential MA intake, and sensitivity to rewarding, aversive, and physiological effects of MA. To further evaluate the causal role of TAAR1 in MA intake, we determined whether replacement of the *Taar1*^*m1J*^ allele with the *Taar1*^*+*^ allele on the MAHDR background, producing MAHDR mice with functional TAAR1, attenuated binge-level MA intake, as it did for MA intake at low concentrations [8].

**Fig. 3:**
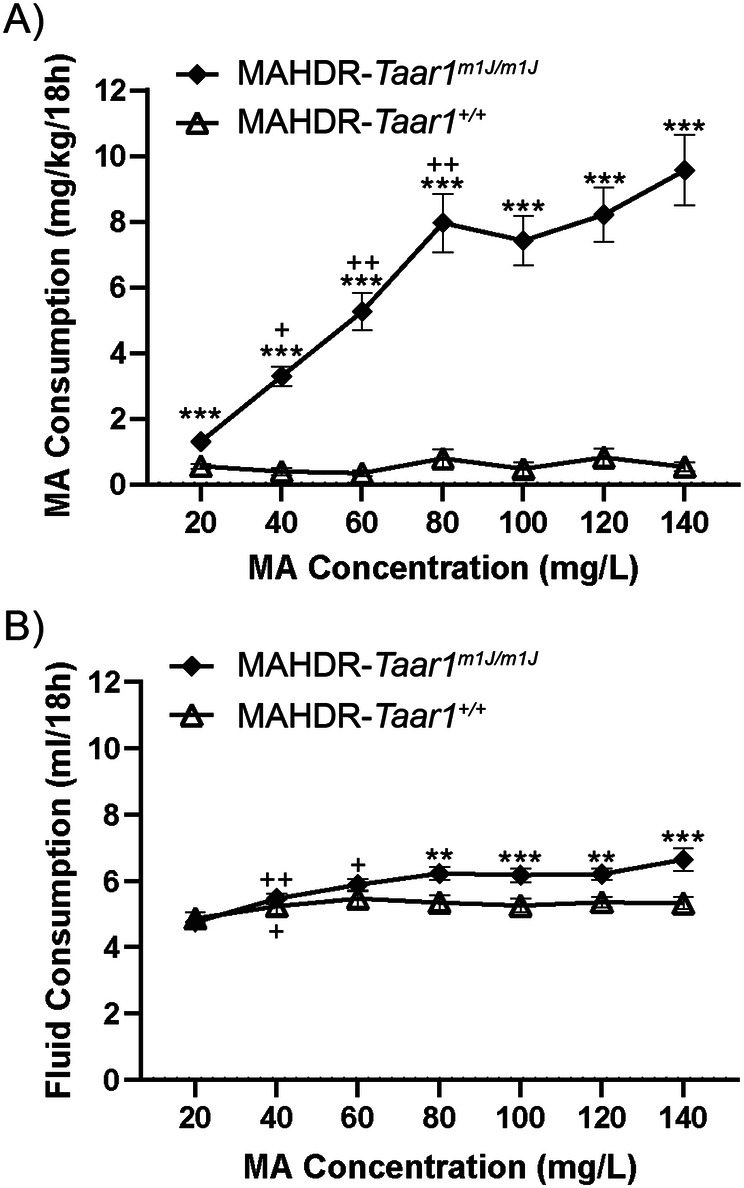
Binge-level MA consumption is dependent on non-functional TAAR1. **A** Total MA (mean ± SEM) consumption in mg/kg/18 h (day 2 and 4 average at each concentration) for each line at each MA concentration offered. **p < 0.01, ***p < 0.001 for the difference in MA consumed between the MAHDR-*Taar1*^*m1J/m1J*^ and MAHDR-*Taar1*^+/+^ KI mice. Further analysis revealed a significant effect of MA concentration for MAHDR-*Taar1*^*m1J/m1J*^ mice only. Within-subjects contrasts of means, ^+^*p* < 0.05, ^++^*p* < 0.01 for the difference in MA consumed compared to the next lower MA concentration. **B** Total fluid (mean ± SEM) consumed by each line during the same 18 h period when each MA concentration was offered. **p < 0.01, ***p < 0.001 for the difference in total volume consumed between the MAHDR-*Taar1*^*m1J/m1J*^ and MAHDR-*Taar1*^*+/+*^ KI lines. Further analysis revealed a significant effect of MA concentration for both the MAHDR-*Taar1*^*m1J/m1J*^ line and MAHDR-*Taar1*^*+/+*^ KI line. Within-subjects contrasts of means, ^+^*p* < 0.05, ^++^*p* < 0.01 for the difference in total volume at a given concentration compared to the previous MA concentration.) *n* = 40 mice (10 mice per line per sex).

No significant effects involving sex for mg/kg MA consumption were found in the initial repeated measures ANOVA, therefore, data were collapsed on sex and reanalyzed for effects of line and MA concentration. There was a significant concentration x line interaction (Fig. 3A; concentration x line: *F*_(6,192)_ = 22.25, *p* < 0.001). MAHDR-*Taar1*^*m1J/m1J*^ mice consumed significantly more MA at all concentrations compared to MAHDR-*Taar1*^*+/+*^ KI mice. Within-subjects contrasts of means between previous and subsequent MA concentration revealed significant increases in MA intake at 40, 60, and 80 mg/L MA concentrations in MAHDR-*Taar1*^*m1J/m1J*^ mice (concentration: *F*_(6,102)_ = 28.18, *p* = <0.001). MAHDR-*Taar1*^*+/+*^ KI mice consumed low and comparable levels of MA at all concentrations offered.

In the initial repeated measures ANOVA for total fluid consumption (ml; MA and water; Fig. 3B) during the 18-h MA access period, there was a significant concentration x sex interaction (*F*_(6,198)_ = 4.27, *p* = <0.001). Total volume consumed by males was significantly greater than females at 140 mg/L. Because there was no interaction of sex with line, data were collapsed on sex and reanalyzed for effects of line and MA concentration. There was a significant concentration x line interaction (concentration x line: *F*_(6,198)_ = 8.15, *p* = <0.001). Total volume consumed by MAHDR-*Taar1*^*m1J/m1J*^ mice was significantly greater than MAHDR-*Taar1*^*+/+*^ KI mice at each concentration between 80 and 140 mg/L. Within-subjects contrasts of means between previous and subsequent MA concentration revealed a significant increase in total volume at 40 mg/L in MAHDR-*Taar1*^*+/+*^ KI mice (concentration: *F*_(6,108)_ = 3.31, *p* = 0.005) and at 40 and 60 mg/L in MAHDR-*Taar1*^*m1J/m1J*^ mice (concentration: *F*_(6,102)_ = 16.69, *p* = <0.001).

### MA-induced potentiation of DR 5-HT neurons is dependent on non-functional TAAR1

**Fig. 4:**
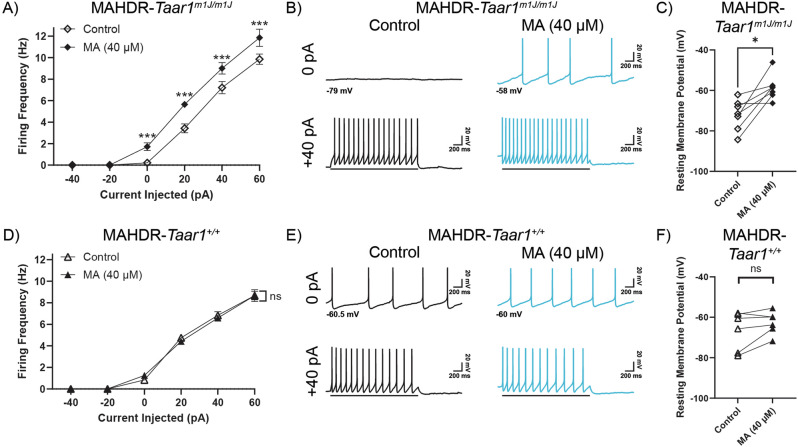
MA-induced potentiation of DR 5-HT neurons is dependent on non-functional TAAR1. Control refers to the firing frequency, activity, and RMP in the presence of antagonists for 5HT_1A_ (WAY 100635, 100 nM) and 5HT_1B_ (SB 216641, 200 nM) autoreceptors. **A** Mean firing frequency of MAHDR- *Taar1*^*m1J/m1J*^ DR 5-HT neurons pre- and post-MA application [7 recordings (7 mice: Male=4, Female=3); Tukey’s HSD post hoc test, ***p < 0.001 for control compared to MA at a given current. Means ± SEM are presented collapsed on sex.] **B** Representative traces of MAHDR-*Taar1*^*m1J/m1J*^ DR 5-HT recordings at 0 pA and +40 pA current injections in the absence (black) and presence of MA (teal). **C** RMPs of MAHDR-*Taar1*^*m1J/m1J*^ DR 5-HT neurons were significantly depolarized by MA, *p < 0.05. Each set of symbols represents a recording in the absence and presence of MA. **D** Mean firing frequency of MAHDR-*Taar1*^*+/+*^ KI DR 5-HT neurons pre- and post-MA application [6 recordings (6 mice: Male=3, Female = 3). Means ± SEM are presented collapsed on sex] **E** Representative traces of MAHDR-*Taar1*^*+/+*^ KI DR 5-HT recordings at 0 pA and +40 pA current injections in the absence (black) and presence of MA (teal). **F** RMPs of MAHDR-*Taar1*^*+/+*^ KI DR 5-HT neurons were unchanged by MA. Each set of symbols represents a recording in the absence and presence of MA.

To determine whether the acute effects of MA on firing frequency of DR 5-HT neurons are dependent on *Taar1* genotype, we compared the effects of MA on DR 5-HT neuron activity of MAHDR-*Taar1*^*+/+*^ KI and MAHDR-*Taar1*^*m1J/m1J*^ mice. DR 5-HT neurons of MAHDR-*Taar1*^*m1J/m1J*^ mice exhibited a significant increase in overall mean firing frequency in response to MA (Fig. 4A; treatment: *F*_(1,6)_ = 28.13, *p* = 0.0018; treatment x current injected: *F*_(5,30)_ = 12.02, *p* = <0.001), similar to MAHDR mice. Representative traces demonstrate the MA-induced potentiation of MAHDR-*Taar1*^*m1J/m1J*^ DR 5-HT neuron activity when recording spontaneous activity and when injecting +40 pA of current (Fig. 4B). MA depolarized the RMPs of DR 5-HT neurons of MAHDR-*Taar1*^*m1J/m1J*^ mice (Fig. 4C; *t*_(6)_ = 3.62, *p* = 0.011). In contrast, mean firing frequency of MAHDR-*Taar1*^*+/+*^ KI DR 5-HT neurons did not change in response to MA across the range of current injections (Fig. 4D, E; *F*_(1,5)_ = 0.029 *p* = 0.87). MA had no effect on RMPs of MAHDR-*Taar1*^*+/+*^ KI DR 5-HT neurons (Fig. 4F; *t*_(5)_ = 1.87, *p* = 0.12).

### MA-induced potentiation of DR 5-HT neuron expressing non-functional TAAR1 is SERT-dependent

**Fig. 5:**
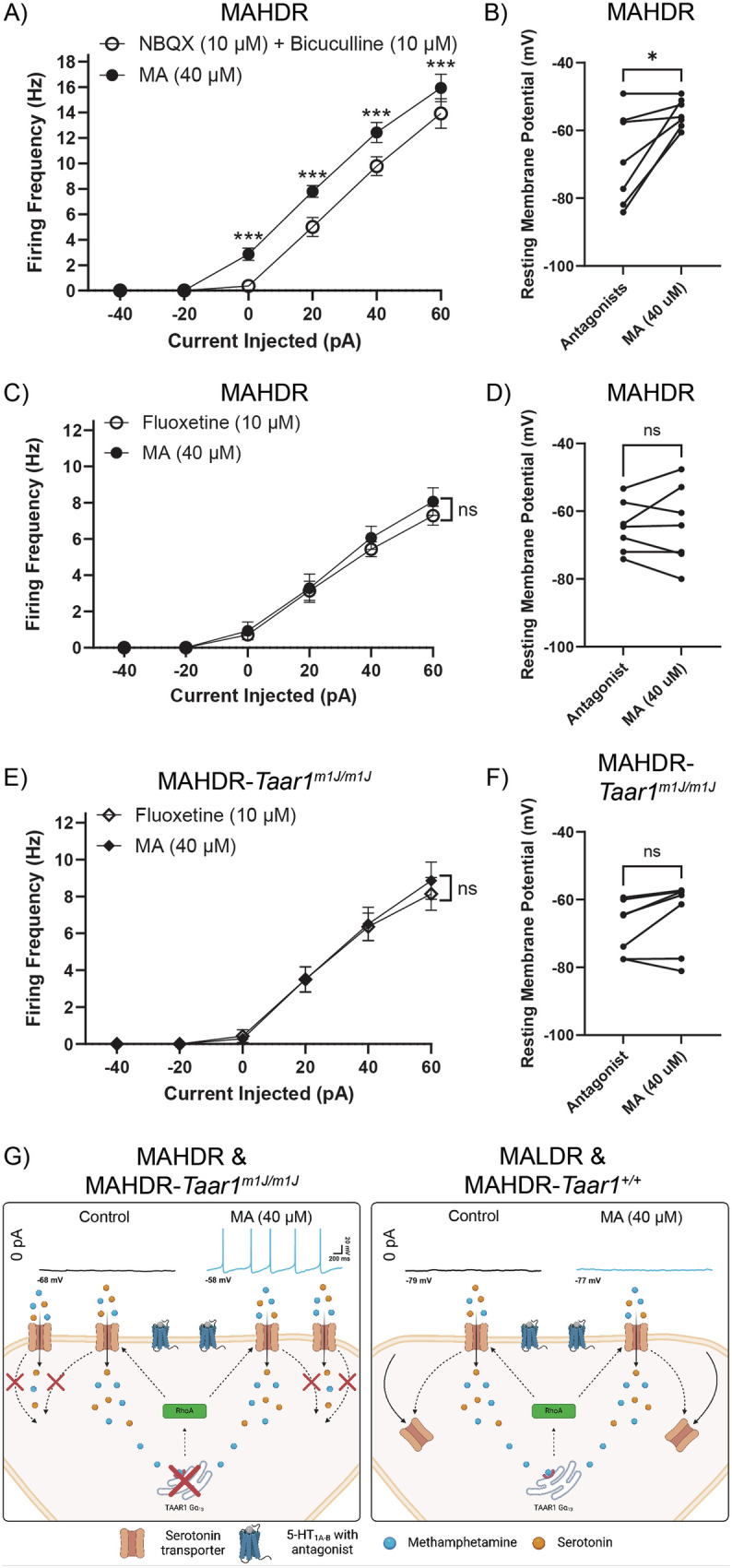
MA-induced potentiation of DR 5-HT neurons expressing non-functional TAAR1 is SERT dependent. All experiments were performed in the presence of antagonists for 5HT_1A_ (WAY 100635, 100 nM) and 5HT_1B_ (SB 216641, 200 nM) autoreceptors. **A** Mean firing frequency of MAHDR DR 5-HT neurons pre- and post-MA application in the presence of the AMPA receptor antagonist NBQX (10 µM) and GABA_A_ receptor antagonist bicuculline (10 µM) [7 recordings (7 mice: Male = 4, Female = 3). Means ± SEM are presented collapsed on sex] **B** RMPs of MAHDR DR 5-HT neurons depolarized in response to MA in the presence of NBQX and bicuculline. Each set of symbols represents a recording in the absence and presence of MA. **C** The effect of MA is blocked in the presence of fluoxetine in recordings from MAHDR DR 5-HT neurons [7 recordings (6 mice: Male=3, Female = 4). Means ± SEM are presented collapsed on sex]. **D** RMPs of MAHDR DR 5-HT neurons were not significantly changed by MA in the presence of fluoxetine. Each set of symbols represents a recording in the absence and presence of MA. **E** Blockade of the MA effect by fluoxetine is confirmed in DR 5-HT neurons from MAHDR-*Taar1*^*m1J/m1J*^ control mice [7 recordings (6 mice: Male = 3, Female = 3). Means ± SEM are presented collapsed on sex]. **F** RMPs of MAHDR-*Taar1*^*m1J/m1J*^ DR 5-HT neurons were not significantly changed by MA in the presence of fluoxetine. Each set of symbols represents a recording in the absence and presence of MA. **G** Schematic of TAAR1 differences between lines. *Left panel:* MA enters DR 5-HT neurons of MAHDR and MAHDR-*Taar1*^*m1J/m1J*^ control mice through SERT. Due to non-functional TAAR1, MA cannot activate TAAR1 signaling pathways, preventing the internalization of SERT from the membrane (denoted by the red Xs). In these mice, MA depolarizes DR 5-HT neurons resulting in increased activity in the presence of autoreceptor antagonists. The effect of MA can be blocked by the SERT antagonist fluoxetine. *Right panel*: MA enters DR 5-HT neurons of MALDR and MAHDR-*Taar1*^*+/+*^ KI mice through SERT. MA activates TAAR1 signaling pathways leading to internalization of SERT from the membrane. As a result, MA does not depolarize DR 5-HT neurons, and their activity remains unchanged in the presence of 5-HT autoreceptor antagonists. Created in BioRender. Rios, S. (2025) https://BioRender.com/t93y794.

To determine whether the TAAR1-dependent MA-induced potentiation observed in DR 5-HT neurons of MAHDR mice is due to synaptic inputs onto DR 5-HT neurons, the AMPA receptor antagonist NBQX (10 µM) and the GABA_A_ receptor antagonist, bicuculline (10 µM) were included in the recording solution, in addition to 5-HT_1A_ and 5-HT_1B_ autoreceptor antagonists. The MA-induced increase in the overall mean firing frequency of DR 5-HT neurons was not blocked (Fig. 5A; treatment: *F*_(1,6)_ = 73.00, *p* = <0.001; treatment x current injected: *F*_(5,30)_ = 12.93, *p* = <0.001). MA significantly increased mean firing frequency throughout the range of injected currents. Additionally, MA depolarized the RMPs of DR 5-HT neurons from MAHDR mice in the presence of NBQX and bicuculline (Fig. 5B; *t*_(6)_ = 3.06, *p* = 0.022). Thus, these synaptic inputs are unlikely to contribute to the MA-induced potentiation and depolarization of DR 5-HT neurons.

Because MA is a substrate of the SERT transporter, we evaluated whether SERT is necessary to observe MA-induced potentiation of DR 5-HT neuron firing in *Taar1*^*m1J/m1J*^ mice. RMPs in the presence of fluoxetine and autoreceptor antagonists were not statistically different compared to RMPs in the presence of autoreceptor antagonists only (MAHDR, *t*_(14)_ = 0.87, *p* = 0.40; N = 7, 9 respectively; MAHDR -*Taar1*^*m1J/m1J*^ controls, *t*_(12)_ = 0.93, *p* = 0.37; N = 7 for both conditions). However, the MA-induced effects on firing frequency and RMP were blocked in the presence of fluoxetine. MA had no effect on mean firing frequency over a range of current injections for MAHDR or MAHDR-*Taar1*^*m1J/m1J*^ DR 5-HT neurons (MAHDR, Fig. 5C; treatment: *F*_(1,6)_ = 2.74, *p* = 0.15; MAHDR-*Taar1*^*m1J/m1J*^, Fig. 5E; treatment: *F*_(1,6)_ = 0.55, *p* = 0.49), indicating SERT is necessary for MA-induced potentiation of DR 5-HT neuron firing. In the presence of fluoxetine, MA had no effect on the RMPs of DR 5-HT neurons of either mouse line (MAHDR, Fig. 5D; *t*_(6)_ = 0.20, *p* = 0.85; MAHDR-*Taar1*^*m1J/m1J*^, Fig. 5F; *t*_(6)_ = 1.92, *p* = 0.10), indicating a role for SERT in MA-induced depolarization. Figure 5G illustrates the hypothesized differences between mice with non-functional TAAR1 (left panel) and functional TAAR1 (right panel).

## Discussion

The data presented show MA-induced depolarization, increases in DR 5-HT neuron excitability, and binge-level MA consumption in mice lacking functional TAAR1. Moreover, MA enhanced DR 5-HT neuron excitability through a SERT-dependent mechanism. These findings highlight the role of TAAR1 as a regulator of DR 5-HT neuron excitability and MA intake.

### KI of functional TAAR1 on the MAHDR background converts binge-level MA intake to low intake

Previously, using MAHDR-*Taar1*^*+/+*^ KI and MAHDR-*Taar1*^*m1J/m1J*^ mice, our lab confirmed a *causal* relationship between *Taar1* genotype and sensitivity to rewarding and aversive effects of MA [11], as well as MA consumption at low MA concentrations [8], which we had *linked* to *Taar1* genotype and TAAR1 function in MADR mice [7, 10]. Building on this, we used the MAHDR-*Taar1*^*+/+*^ KI and MAHDR-*Taar1*^*m1J/m1J*^ mice to determine whether TAAR1 function is critical for binge-level MA consumption, as observed in MAHDR mice [12]. Binge-level MA consumption was found in MAHDR-*Taar1*^*m1J/m1J*^, but not MAHDR-*Taar1*^*+/+*^ KI mice. Therefore, KI of *Taar1*^*+*^, which expresses functional TAAR1, converted binge-level MA intake to low MA intake, confirming that *Taar1 causally* regulates MA consumption and the presence of non-functional TAAR1 drives binge-level MA intake. Given TAAR1’s critical role in determining MA consumption and sensitivity to MA reward, we explored whether TAAR1 function determines the effects of MA on DR 5-HT neurons, which are involved in reward signaling.

### TAAR1 functionality mediates MA-induced changes in DR 5-HT neuron activity

MA inhibited firing of MALDR DR 5-HT neurons that have functional TAAR1. This inhibition is consistent with studies using other TAAR1 agonists [24, 29]. MA and other psychostimulants increase extracellular 5-HT concentrations which activates inhibitory 5-HT autoreceptors [18, 19, 23, 30, 31]. Our findings support this mechanism, as MA alone hyperpolarized MALDR DR 5-HT neurons, and both the hyperpolarization and inhibition of firing were reversed by 5-HT autoreceptor antagonists. In contrast, MA depolarized and increased the firing of DR 5-HT neurons in the presence of 5-HT autoreceptor antagonists in recordings from mice with non-functional TAAR1 (MAHDR and MAHDR-*Taar1*^*m1J/m1J*^). Importantly, the MA-induced depolarization and increase in excitability were absent in MAHDR-*Taar1*^*+/+*^ KI which are genetically matched to the MAHDR-*Taar1*^*m1J/m1J*^ mice except for the single point mutation in TAAR1. This indicates that lack of TAAR1 functionality is crucial for MA-induced depolarization and excitability of DR 5-HT neurons, likely contributing to heightened reward sensitivity. The effect of MA on the firing rate and RMP of MAHDR DR 5-HT neurons was maintained in the presence of AMPA and GABA_A_ receptor antagonists, suggesting these synaptic inputs do not mediate the effect of MA. This does not rule out other neuromodulators that may be present in our slices that could mediate the depolarization, such as norepinephrine [26, 32].

### MA-induced depolarization and excitability of DR 5-HT neurons expressing non-functional TAAR1 is SERT dependent

The next set of experiments identified SERT as the cellular mechanism underlying the MA-induced increase in DR 5-HT neuron excitability. Fluoxetine completely blocked the effects of MA on RMP and excitability of DR 5-HT neurons from MAHDR and MAHDR-*Taar1*^*m1J/m1J*^ mice. MA, as a substrate of SERT [26], is transported into DR 5-HT neurons, where it can interact with TAAR1 as a potent agonist [9]. Notably, our previous research described amphetamine-induced internalization of DAT in midbrain dopamine neurons [33, 34] via TAAR1-dependent G_13_-mediated RhoA signaling [35]. Similarly, TAAR1-mediated internalization of SERT has also been described, which is significant because SERT expression on the membrane tightly regulates extracellular 5-HT levels [36]. In DR 5-HT cultures expressing functional TAAR1, the psychostimulant MDMA activates TAAR1 leading to increased G_13_-coupled RhoA signaling. This cascade drives SERT internalization, reducing serotonin reuptake [23]. Importantly, MDMA has no effect on the surface expression of SERT in TAAR1 KO cells [23]. These results indicate that MDMA, a derivative of MA, interacts with both SERT and TAAR1. Since we observed MA-induced increases in firing in DR 5-HT neurons from mice that have non-functional TAAR1, it is likely that TAAR1 is unable to traffic SERT from the membrane, allowing for the continued transport of MA. One possible mechanism explaining the depolarization and increase in excitability of DR 5-HT neurons is an increase in SERT-dependent ion currents. Amphetamine and MA stimulate DAT-dependent currents that are uncoupled from electrogenic transport and increase the excitability of DA neurons [37, 38]. SERTs expressed in cell lines also display coupled and uncoupled ion currents [39–42]. The SERT-dependent increase in excitability of DR 5-HT neurons from mice with non-functional TAAR1 suggests that impaired internalization of SERT may reveal MA-induced SERT currents, which would be responsible for the observed MA-induced depolarization that is blocked in the presence of fluoxetine. We cannot rule out the possibility that maintaining SERT on the membrane also allows MA transport into the neurons to affect ion channels from the intracellular space or activate ion channels downstream of TAAR1 signaling. Further experiments are necessary to determine the discrete site where MA interacts to increase excitability.

In summary, we observed MA-induced depolarization and an increase in the firing frequency of DR 5-HT neurons in MA reward-sensitive MAHDR and MAHDR-*Taar1*^*m1J/m1J*^ mice and observed binge-level MA intake by MAHDR-*Taar1*^*m1J/m1J*^ mice but not MA reward-insensitive MAHDR-*Taar1*^*+/+*^ mice. These data further support a critical link between DR 5-HT neuron activity and reward-related behaviors. The dual dependence of MA-induced changes in RMP and the firing rate of DR 5-HT neurons on both TAAR1 functionality and SERT activity further underscores a complex regulatory mechanism. This intricate relationship highlights the necessity for further investigation into how TAAR1 and SERT interact to modulate DR 5-HT neuron activity in response to MA, particularly considering the dramatic effect the *Taar1* mutation has on MA consumption [7, 8, 11]. Exploring this interplay will provide insights into the genetic and molecular mechanisms influencing MA use disorders and uncover novel therapeutic targets.

## Data Availability

All data are available upon reasonable request to the authors.
